# Follow-up of participants with subjective cognitive decline from Tremembé epidemiologic study, Brazil

**DOI:** 10.1590/1980-5764-DN-2022-0064

**Published:** 2023-05-29

**Authors:** Karolina Gouveia César-Freitas, Ana Catarina Penalva Berardis, Thaísa Valentim Moreira Pretto, Amanda Monteiro Viagi, Vitorio Lourençon, Leonardo Yuri Kasputis Zanini, Isabella Caroline Callegari Barbosa, Rubya Pasquarelli Machado, Natália Gomes Manso Cunha, Maria Júlia Lumi Watanabe, Mario Amore Cecchini, Sonia Maria Dozzi Brucki, Ricardo Nitrini

**Affiliations:** 1 Universidade de São Paulo, Faculdade de Medicina, Unidade de Neurologia Cognitiva e Comportamental, Departamento de Neurologia, São Paulo SP, Brazil.; 2 Universidade de Taubaté, Departamento de Medicina, Taubaté SP, Brazil.

**Keywords:** Incidence, Dementia, Epidemiology, Cognitive Dysfunction, Incidência, Demência, Epidemiologia, Disfunção Cognitiva

## Abstract

Subjective cognitive decline is defined as a self-perceived cognitive decline but with normal performance in neuropsychological assessments.

**Objective:**

To verify the evolution of patients diagnosed with subjective cognitive decline compared to the cognitively normal group without any concern.

**Methods:**

This is a follow-up study based on data analysis from the Tremembé epidemiologic study, in Brazil. The 211 individuals classified as cognitively normal and 174 diagnosed as having subjective cognitive decline at baseline were invited to participate.

**Results:**

After a median follow-up time of five years, 108 subjective cognitive decline participants (62.0%) were reassessed. Of these, 58 (53.7%) kept this diagnosis, whereas 14 individuals (12.9%) progressed to mild cognitive impairment and 5 (4.6%) to dementia. In the cognitively normal group, 107 (50.7%) were reassessed, of which 51 (47.7%) were still classified likewise, 6 (5.6%) evolved to mild cognitive impairment and 9 (8.4%) to dementia. The presence of cognitive decline had a significant association with increasing age and depression symptoms. Considering the total number of baseline participants in each group: the subjective cognitive decline group showed higher percentage of mild cognitive impairment (p=0.022) and no difference was found in progression to dementia (p=0.468) between the groups after follow-up assessment.

**Conclusion:**

Most subjective cognitive decline participants at baseline kept their cognitive complaint at follow-up and this group progressed more to mild cognitive impairment than the other group. No difference in the progression to dementia was found, despite the higher incidence of dementia in the cognitively normal group.

## RESUMO.

O declínio cognitivo subjetivo (DCS) é definido como autopercepção de declínio cognitivo, mas com desempenho normal nas avaliações neuropsicológicas.

**Objetivo:**

O objetivo foi verificar a evolução dos pacientes diagnosticados com DCS em relação ao grupo cognitiva mente normal (CN), sem qualquer queixa.

**Métodos:**

Trata-se de um estudo de seguimento baseado na análise de dados do estudo epidemiológico de Tremembé, Brasil. Os 211 indivíduos classificados como CN e os 174 diagnosticados como DCS na fase inicial do estudo foram convidados a participar.

**Resultados:**

Após o tempo médio de seguimento de cinco anos, 108 participantes da DCS (62,0%) foram reavaliados. Deles, 58 (53,7%) mantiveram o diagnóstico de DCS, enquanto 14 (12,9%) evoluíram para comprometimento cognitivo leve (CCL) e cinco (4,6%) para demência. No grupo CN, 107 (50,7%) foram reavaliados, dos quais 51 (47,7%) ainda foram classificados como CN, seis (5,6%) evoluíram para CCL e nove (8,4%) para demência. A presença de declínio cognitivo teve associação significativa com o aumento da idade e com sintomas de depressão. Considerando-se o número total de participantes da fase inicial do estudo de cada grupo: o grupo DCS apresentou maior percentual de CCL (p=0,022) e não houve diferença na progressão para demência (p=0,468) entre ambos os grupos após a avaliação de seguimento.

**Conclusão:**

A maioria dos participantes DCS da fase inicial do estudo manteve sua queixa cognitiva no seguimento, e esse grupo progrediu mais para CCL. Não foi encontrada diferença na progressão para demência, apesar da maior incidência de demência no grupo CN.

## INTRODUCTION

Cognitive impairment is one of the major health problems due to the growth of the elderly population, and therefore, the early diagnosis of dementia has been the goal of several studies^1^. Currently, studies indicate that subjective cognitive decline (SCD), characterized by the self-experience of deterioration in cognitive performance not detected objectively through formal cognitive assessments, may be an early marker of dementia^2^. Epidemiological data have shown that individuals with SCD may be at an increased risk of progression to dementia. However, Alzheimer's disease may not be the only cause, and several other conditions can be associated with this condition, from neuropsychological disorders to normal aging^3^.

Mild cognitive impairment (MCI) is defined by the presence of complaints reported by the patient and/or informant along with evidence through objective neuropsychological evaluation (usually with scores 1.5 standard deviation below the normative mean for education level), with the preservation of independence in daily activities, and that does not meet the criteria for dementia^4,5^. Cognitive impairment no dementia (CIND), another condition between normal ageing and dementia state, is a more comprehensive term and can be considered for individuals with cognitive performance lower than expected for age and education level; however, the evidence of a decline or progression is not essential^6,7^. Therefore, to summarize: the SCD individuals have the complaint, but their cognitive evaluation is entirely within the normal range; the MCI individuals, in addition to the complaint by themselves and/or informant, have cognitive impairment with lower performance than expected in the cognitive tests; and the CIND individuals have impairment evidence in the neuropsychological assessments as MCI, but without confirmation of decline by themselves or informant.

A Mayo Clinic study revealed a prevalence of SCD between 12.3% and 57% among 1,167 cognitively healthy participants aged between 70 and 95 years^8^. In that study, it was noticed that the concern with memory was present in 24% of the population, and it is the greatest predictor of the incidence of MCI when compared to other cognitive domains, such as language, visual-spatial skills, planning, organization, and attention^8^. In the same study, the authors showed that about 12% of SCD patients evolved to MCI during a median follow-up of 3.9 years^8^.

The present study aimed to verify the evolution of participants who were diagnosed with SCD at baseline of Tremembé epidemiologic study (TES) and to identify if they had a higher incidence of MCI or dementia than individuals diagnosed as cognitively normal (CN).

## METHODS

The baseline study was carried out in Tremembé municipality in São Paulo state, Brazil, in which home visits were conducted with 20% of the population aged 60 years and over through a random selection^9^. At the baseline, participants underwent history taking, physical and neurological examination, cognitive assessment, psychiatric evaluation, and functional activity questionnaires. Afterward, they were classified into normal cognition (n=385), CIND (n=135) and those with dementia (n=110). The dementia prevalence was of 17.5% (95% confidence interval [CI] 14.6–20.6)^9^.

All participants without dementia at baseline were invited to the follow-up study within up to five years, and the incidence rate of dementia was 22.3 per 1,000 person-years (95%CI 17.1–48.8/1,000 person-years)^10^. Individuals classified as normal cognition at baseline were divided into two groups: cognitively normal (CN) without cognitive complaints (n= 211) and those with SCD (n= 174) to compare their cognitive evolution in this cohort^9,10^. A community health agent contacted all the 385 participants, inviting them to schedule a new home visit. Participants who refused to participate in the follow-up visit, or those who were not living in the same address or could not be reached by telephone were excluded.

The assessment was done by the first author (KGCF) and nine medical graduate students at the University of Taubaté (co-authors), who had previously been trained to perform the cognitive tools and the exam protocol. The participants or legal guardians were fully informed about the study and signed a consent form, as approved by the Ethics Committee of the University of São Paulo and by the University of Taubaté.

### Assessment

The participants who agreed to participate were submitted to clinical and cognitive evaluations. A brief clinic history was performed by investigating the presence of cognitive decline complaints, medications in use, and some new health information or event during the period between the first and the second assessment. Subjective complaints were assessed by asking the participant directly: “Do you feel that your memory is getting worse?” and to the informant: “Does your relative have any recent memory problem?”. In addition, blood pressure was measured, besides the application of the cognitive tests, functional activity questionnaire, and psychiatric scale, all in a single home visit. The neuropsychological evaluation comprised: Mini-Mental State Examination (MMSE)^11,12^, Brief Cognitive Screening Battery (BCSB)^13,14^, and phonemic verbal fluency test (letter P). The Functional Activities Questionnaire (FAQ)^15^ was answered by the informants or a family member and the Cornell Scale for Depression in Dementia^16,17^ was applied to quantify depressive symptoms.

The MMSE version used was the validated in Portuguese and the cutoff points were adapted according to the educational level^12^. The BCSB assessed the visual perception and the naming of ten black and white drawings shown on a sheet of paper and, subsequently, the learning and recalling of these figures after exhibiting three times (for testing incidental memory, immediate memory, learning, and delayed recall). In the necessary interval between learning and delayed recall, semantic verbal fluency (animals) and the clock drawing test were applied^13,14,18^.

The FAQ scores ranged from 0 to 30 points; and a score greater than or equal to 5 was associated with the presence of dementia^15^. Although the Cornell scale was originally developed for the diagnosis of depression when the score was greater than or equal to 8 points in patients with dementia, it is an instrument that can be used on geriatric subjects with or without dementia^16,17^.

Therefore, the diagnosis of depression was based on the Cornell scale score. Though, other diseases such as hypertension, diabetes, and stroke were identified in the clinical history through self-report and corroborated with additional information from the physical examination and list of medication in use.

### Clinical diagnoses

The clinical diagnoses were established in consensus meetings by KGCF and other neurologists specialized in cognitive neurology (two last authors, SMDB and RN) based on the discussion of all evaluations done in this phase of the study compared to the baseline evaluation. Only KGCF knew which participant had a complaint at baseline. After that, the participants were classified as CN, SCD, CIND, MCI or dementia based on the criteria reported below.

For the diagnosis of CN, the participant should have a score equal to or greater than those defined according to the educational level. The MMSE cutoff scores were: for illiterate, 20 points; for 1 to 4 years of education, 25 points; for 5 to 8 years, 27 points; for 9 to 11 years, 28 points; and for individuals with more than 11 years, 29 points^12^. For the delayed recall of the BCSB, the cutoff score was 6 points^13^. For the semantic verbal fluency test, the cutoff scores were 9 for illiterates, 12 for individuals with 1 to 7 years of education, and 13 for those with 8 or more years of education^19^. Besides the numerical scores, for the tests of clock drawing and phonemic verbal fluency, a qualitative comparison were used between the first score at the baseline phase with the second evaluation; once these tests are strongly influenced by educational level, the decline was considered.

The diagnostic criteria for SCD was based on the presence of cognitive complaint, either with normal or higher performance in cognitive scores, as described above^3^. As for CIND, it was based on the performance in cognitive tests (memory and/or other cognitive domains) below education-adjusted cutoff scores, absence of a cognitive complaint, and lack of functional impairment (score less than 5 in FAQ)^20^. For MCI, it was based on the presence of a cognitive complaint and in accordance with the performance in cognitive tests (memory and/or other cognitive domains) of 1.5 standard deviation below education-adjusted cutoff scores, and absence of functional impairment (score less than 5 in FAQ)^5^. Dementia was diagnosed based on clinical criteria by the National Institute on Aging – Alzheimer's Association (NIA-AA) and according to the criteria of McKhann et al.^21^. For the diagnosis of dementia, the participant should have cognitive complaint, clinical history consistent with cognitive decline, cognitive tests below education-adjusted cutoff scores in at least two cognitive domains or one cognitive domain plus behavior, and score equal to or greater than 5 in FAQ.

### Statistical analysis

The statistical analysis was performed using the Statistical Package for the Social Sciences (SPSS) version 25.0 for Windows. The CN and SCD diagnostic groups were compared considering data from baseline: age, education, diseases such as depression, hypertension, diabetes, and stroke. As the variables age and education did not follow normal distribution in all groups, the Mann-Whitney test was used. The comparison of the follow-up groups was conducted using the Mann-Whitney test for continuous variables, such as age, education, and Cornell score. Subsequently, a multiple comparison test (Dunn-Bonferroni's post hoc correction) was applied in order to verify the differences among stable participants, those who progressed to CIND, MCI or dementia. Categorical variables, such as depression, diabetes, hypertension, and stroke were compared using the Pearson's chi-square test. For all analyzes, a significant p-value <0.05 was adopted.

## RESULTS

**Figure 1:**
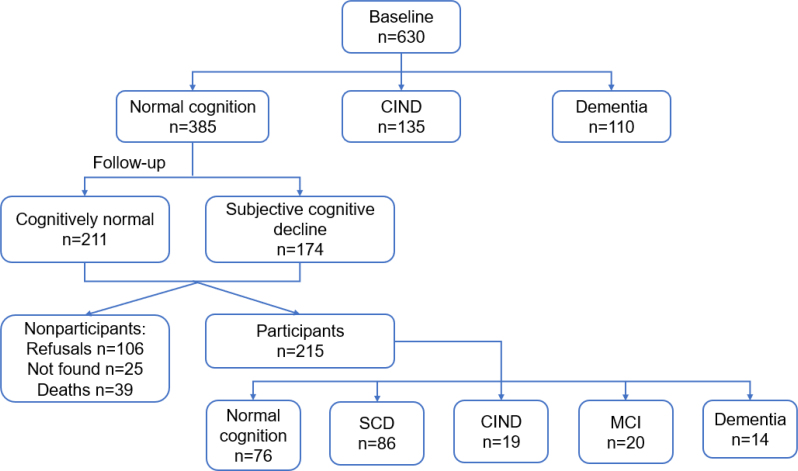
Flowchart of study participants.

From the 385 individuals classified as normal at baseline, 215 were reassessed in this study. The reasons for not participating in this phase of the study were: 106 refused follow-up, 39 deceased, and 25 changed address and were not found even by phone (Figure 1). The 215 participants were reclassified after follow-up reassessment into: CN, SCD, CIND, MCI, and dementia. The demographic characteristics of the sample at baseline was shown in Table 1. The median follow-up time was 5 years (2–6 years).

**Table 1:** Characteristics of the sample by cognitive status at baseline (n=385).

Age (years), mean (SD)		Cognitively normal (n=211)	Subjective cognitive decline (n=174)	p-value
		69.9 (7.3)	68.5 (6.6)	0.073*
Female, n (%)		128 (60.7)	125 (71.8)	0.022†
Race, n (%)	White	156 (73.9)	133 (76.4)	0.316†
	Black	54 (25.6)	38 (21.8)	
	Asian	1 (0.5)	3 (1.7)	
Education (years), mean (SD)		5.2 (4.8)	6.1 (4.8)	0.011*
Diabetes, n (%)		63 (29.9)	50 (28.7)	0.810†
Hypertension, n (%)		149 (70.6)	121 (69.5)	0.818†
Coronary artery disease, n (%)		15 (7.1)	22 (12.6)	0.067†
Stroke, n (%)		3 (1.4)	8 (4.6)	0.063†
Psychiatric disorder, n (%)		51 (24.2)	74 (42.5)	<0.001†
Smoking, n (%)	Never	164 (77.7)	146 (83.9)	0.291†
	Current	29 (13.7)	16 (9.2)	
	Former	18 (8.5)	12 (6.9)	
Alcohol use, n (%)	No use or light use	161 (76.3)	143 (82.2)	0.143†
	Moderate use	37 (17.5)	23 (13.2)	
	Current or previous abuse	13 (6.2)	8 (4.6)	

Abbreviations: SD: standard deviation.

* Notes: Mann-Whitney Test;

† Pearson's Chi-square Test.

The participants were grouped according to the diagnosis received at baseline: 107 of the 211 participants in the CN group were re-evaluated and 108 were re-evaluated in the second group of 174 participants diagnosed with SCD, as shown in Table 2. The groups were similar regarding the percentage of non-participation: deaths (p=0.116), refusals (p=0.505), and people not found (p=0.170). It was observed in the CN group that most participants remained with the cognitive condition unchanged, maintaining the CN diagnosis (p=0.016); the same occurred in the group diagnosed as SCD at baseline, the participants remained mostly with the same diagnosis (p<0.001). Considering the total number of baseline participants in each group: the baseline SCD group showed higher percentage of MCI (p=0.022) and no difference was found in progression to dementia (p=0.468) between both group after follow-up assessment.

**Table 2:** Classification of the two groups of participants after follow-up assessment.

Follow-up	Cognitively normal (%)	Subjective cognitive decline (%)	p-value*
Deaths	26 (12.3)	13 (7.4)	0.116
Refusals	61 (28.9)	45 (25.9)	0.505
Not found	17 (8.0)	8 (4.6)	0.170
CN	51 (24.1)	25 (14.3)	0.016
SCD	28 (13.2)	58 (33.3)	<0.001
CIND	13 (6.1)	6 (3.4)	0.221
MCI	6 (2.8)	14 (8.0)	0.022
Dementia	9 (4.3)	5 (2.9)	0.468
Total	211	174	

Abbreviations: CN: cognitively normal; SCD: subjective cognitive decline; CIND: cognitive impairment no dementia; MCI: mild cognitive impairment.

* Note: Pearson's Chi-square Test. Bold numbers indicates statistical significance.

Therefore, the percentages presented in Table 2 refer to the total of each baseline group. However, when the number of cases did not consider the nonparticipants, the value of conversion to MCI at baseline changed to 5.6% in the CN group and 12.9% in the SCD group (p=0.063). Likewise, the corrected conversion rate to dementia in the CN group was 8.4% and in the SCD group was 4.6% (p=0.261). Besides that, analyses were performed comparing age, education and MMSE score between participants that did and did not attend to the follow-up evaluation. The results showed no significant differences between the groups who did and did not attend the second evaluation: in the CN group, they had similar age (p=0.312), education (p=0.322), and MMSE score (p=0.509); in the SCD group they also had similar age (p=0.071), education (p=0.685) and MMSE (p=0.308). Age was close to reach significance level in the SCD group; however, the effect size was small (d=0.282), showing no significant differences between the groups.

The participants were compared according to the baseline diagnosis, verifying some variables considered as possible factors associated with the cognitive decline (Table 3). The SCD individuals had a higher prevalence of depression, therefore, a higher mean score on the Cornell scale and a higher occurrence of stroke. The two group was similar in relation to age and education level.

**Table 3:** Possible variables associated with cognitive impairment distributed according to the subjective cognitive decline group and the cognitively normal group at baseline after the follow-up assessment.

Variables	Cognitively normal (n=107)	Subjective cognitive decline (n=108)	p-value
Age (years)	69.36 (±6.64)	67.81(±5.85)	0.099*
Education (years)	5.52 (±5.13)	6.23 (±4.97)	0.123*
Cornell scale	5.64 (±4.69)	8.21(±5.83)	<0.001*
Depression	29 (27.1%)	54 (50.0%)	0.001†
Diabetes	29 (27.1%)	34 (31.5%)	0.481†
Hypertension	77 (72.0%)	76 (70.4%)	0.797†
Stroke	1 (0.9%)	7 (6.5%)	0.032†

Abbreviations: CN: cognitively normal; SCD: subjective cognitive decline.

* Notes: Mann-Whitney Test;

† Pearson's Chi-square Test.

Bold numbers indicates statistical significance.

Table 4 shows in the ten columns the reclassifications of the participants. The column representing CN–CN, for example, means that the individuals who had the diagnosis of CN at the baseline study, remained as CN in the follow-up evaluation. That is to say that the first initials represent the diagnosis at baseline and the second ones, separated by a hyphen, refer to the follow-up diagnosis. Another example, the column SCD–MCI refers to data from SCD participants at baseline who progressed to MCI in the follow-up. It is noteworthy that after finding the significant difference in age, Cornell scale and presence of depression, post hoc analysis was performed for continuous variables, which were represented by the letters where there was significance (see Table 4 legend). It can also be noted that in relation to the baseline group classified as CN: six that evolved to MCI had higher rates of depression and higher mean score of Cornell scale; and the nine individuals who progressed to dementia had a higher mean age (p=0.009), and although not significant, the dementia subgroup had lower mean education, and a higher percentage of hypertension. Regarding the group initially classified as SCD at baseline, the progression to MCI was present in 14 patients who, even not significant, had higher rates of chronic diseases and previous stroke; and the five individuals who progressed to dementia presented high mean age (p=0.009). Furthermore, the 25 SCD participants at baseline had the diagnosis changed to CN (p=0.016), which means they did not keep cognitive complaints in follow-up, probably due to a lower percentage of depression (p<0.001) in this group.

**Table 4:** Analysis of factors associated with cognitive impairment such as mean age, education (in years), mean Cornell score, and percentage of depression, diabetes, hypertension, and of stroke, within the subgroups classified as CN and SCD at baseline with participants’ diagnosis after follow-up.

Variables	CN-CN (n=51)	CN-SCD (n=28)	CN-CIND (n=13)	CN-MCI (n=6)	CN-Dementia (n=9)	SCD-CN (n=25)	SCD-SCD (n=58)	SCD-CIND (n=6)	SCD-MCI (n=14)	SCD-Dementia (n=5)	p-value
Age	68.69 (±6.50)^a^	67.29 (±4.81)	72.23 (±6.25)	69.17 (±8.54)	75.67 (±7.79)^b^,^c^	66.72 (±5.71)^d^	67.14 (±5.17)	70.33 (±4.84)	69.36 (±6.06)	73.80 (±10.62)	0.009*
Education	5.00 (±4.98)	7.86 (±5.92)	4.85 (±3.16)	5.00 (±5.18)	2.56 (±3.47)	7.36 (±6.29)	5.78 (±4.79)	4.50 (±3.27)	7.14 (±3.80)	5.40 (±3.85)	0.066*
Cornell	5.06 (±4.32)^e^	4.07 (±2.79)^e^	7.31 (±4.84)	12.00 (±8.08)	7.11 (±4.91)	5.76 (±4.57)^e^	9.36 (±5.94)^b^,^f^	9.17 (±2.56)	8.86 (±7.57)	4.20 (±2.78)	<0.001*
Depression	12 (23.5%)	4 (14.3%)	4 (30.8%)	5 (83.3%)	4 (44.4%)	10 (40.0%)	35 (60.3%)	4 (66.7%)	5 (35.7%)	0	<0.001†
Diabetes	11 (21.6%)	8 (28.6%)	5 (38.5%)	2 (33.3%)	3 (33.3%)	7 (28.0%)	18 (31.0%)	1 (16.7%)	6 (42.9%)	2 (40.0%)	0.897†
Hypertension	32 (20.9%)	21 (75.0%)	11 (84.6%)	5 (83.3%)	8 (88.9%)	20 (80.0%)	40 (26.1%)	4 (66.7%)	10 (71.4%)	2 (40.0%)	0.491†
Stroke	1 (2.0%)	0	0	0	0	1 (4.0%)	3 (5.2%)	0	3 (21.4%)	0	0.075†

Abbreviations: CN: Cognitively normal; SCD: subjective cognitive decline; CIND: cognitive impairment no dementia; MCI: Mild cognitive impairment; the diagnoses are separated by a hyphen, indicating the diagnose on the first and the second assessment.

* Notes: Kruskal Wallis test:

a differs from CN–Dementia;

b differs from CN–CN;

c differs from SCD–Dementia;

d differs from SCD–MCI;

e differs from SCD–SCD;

f differs from CN–MCI;

† Pearson's Chi-square Test.

Bold numbers indicates statistical significance.

## DISCUSSION

Participants of the SCD group at baseline had a significant higher incidence of MCI than the CN group, considering the nonparticipants, 14 (12.9%) and 6 (5.6%) cases, respectively, at follow-up. Notwithstanding, the corrected conversion rate to MCI excluding the number of nonparticipants was almost significant (p=0.062). Moreover, there was no significant difference between both groups regarding incidence of dementia, although there was a higher number of dementias in the CN group compared to the SCD group, 9 (8.4%) and 5 (4.6%) cases, respectively. Excluding or not the number of nonparticipants, the progression rate to dementia in both groups maintained as nonsignificant, probably due to the small sample size in each subgroup. The participants who evolved to dementia after a median of five years from the first evaluation were the oldest (p=0.009), when analyzing the potential risk factors within all subgroups.

The Mayo Clinic study described that 14% of participants with SCD evolved to MCI, similar to the data found in our research, in which 12.9% of individuals with SCD evolved to MCI^8^. Although there is a possibility that SCD represents a possible pre-symptomatic stage for MCI and Alzheimer's disease, the time for the conversion has been variable; SCD has been reported 15 years before MCI^22,23^. Therefore, the 5 year follow-up time of our study may have not been enough to observe dementia conversion. Besides, the follow-up time enough to detect cognitive impairment varies in the literature, from 3.9 to 15 years, and some authors suggested that the decline in most subjects could have been perceived 7-years mean before conversion to MCI^8,22,24^. Nevertheless, many participants may have already had the cognitive complaint for some time before baseline.

There was a significant association between age and depressive symptoms with the evolution to the diagnosis of cognitive impairment — whether MCI or dementia — and individuals who developed dementia were the eldest. Though, a significant association was not found with years of education and with chronic diseases. Despite the level of education not being statistically significant, when observing the means, we found lower values in individuals who presented any type of cognitive impairment. This finding could be observed in all comparisons, except in the baseline SCD group that evolved to MCI who had 7.14 years of mean education. Even with high education level, in the subgroup SCD that progresses to MCI, a greater proportion of hypertension and diabetes was found considering other groups, even not significant, probably due to the small sample size of the subgroups.

Other studies suggest that SCD may be more associated with non-neurodegenerative causes, such as depressive symptoms, anxiety, certain personality traits, or failing physical health, corroborating our findings of significant correlation with depression^25,26^. The presence of hypertension and diabetes had no significant association with cognitive impairment in this study, probably due also to the small sample in some subgroups. However, data from the literature have shown the correlation between stroke and cognitive decline, as well as factors that have been widely confirmed as low education and increasing age^7,27^. Although it was not a significant finding, some comorbidities were present in all subgroups analyzed – in a lower percentage in the individuals who maintained the CN condition and in a higher percentage in those who were CN and progressed to dementia. The SCD individuals at baseline who evolved to MCI at follow-up had the highest percentage of diabetes.

The evolution of CN group at baseline showed a higher diagnosis of individuals with dementia and those with SCD had a higher diagnosis of MCI. Even though a greater incidence of both diagnoses within the SCD group was expected, since this group may be associated with a higher risk of degenerative disease, it can also be associated with other causes. The complaint reported may be of memory or other cognitive function and this is quite complex. Although the predominant concern was about memory, it is already a heterogeneous phenomenon. It is known that multimorbidity, polypharmacy, greater use of health services, pain and poor self-perceived health are associated with memory complaint^25^.

In addition, the biomarkers features should determine which SCD subjects could be classified as NIA-AA stage 2 if positive for amyloid and predict the evolution to objective cognitive decline, dementia, and Alzheimer's disease^28,29^. As SCD could be a very heterogeneous entity, some features have been appointed as decline risk: onset of subjective decline within 5 years, confirmation of cognitive decline by an informant, and decline-related worries besides of lower amyloid Aß-42 levels, and/or hyperphosphorylated tau changes and/or neurodegeneration presence fulfilling the ATN criteria^30–32^. Nevertheless the biomarkers use is not available in clinical practice yet, highlighting the importance of the cognitive assessment.

This study has limitations, such as the high non-participation rate at the follow-up, the small sample size in each subgroup, and the absence of biomarkers and neuroimaging to confirm the diagnosis. Regarding the small number of individuals in the subgroups, it is important to observe that the initial number of participants was proportional to the Tremembé elderly population census data^9^. This study did not aim at a more detailed statistical analysis and with adjusted logistic regression models, since the incidence of dementia in this cohort with these analyzes had already been published^10^. Therefore, this study is more for qualitative evaluation of the cognitively normal groups at baseline.

Some strengths of this study are worth mentioning. The use of the same cognitive instruments at the baseline facilitated the comparison between the two assessments. The cutoff scores were defined according to educational level, making the diagnosis more trustworthy. The home assessments were done by only nine trained assessors, minimizing the bias among examiners. Moreover, the diagnoses were established by consensus among three neurologists with expertise in epidemiological studies of dementia in Brazil.

In sum, this study has clinical importance, since it is a population-based study in a middle-income country, which is scarce. The longitudinal studies could allow us to establish the incidence of certain diseases, creating prevention measures and changing policies of public health.
